# How do primary health care teams learn to integrate intimate partner violence (IPV) management? A realist evaluation protocol

**DOI:** 10.1186/1748-5908-8-36

**Published:** 2013-03-23

**Authors:** Isabel Goicolea, Carmen Vives-Cases, Miguel San Sebastian, Bruno Marchal, Guy Kegels, Anna-Karin Hurtig

**Affiliations:** 1 Unit of Epidemiology and Global Health, Department of Public Health and Clinical Medicine, Umea University, SE-90187 Umea, Sweden; 2 Public Health Research Group, Department of Community Nursing, Preventive Medicine and Public Health and History of Science, Alicante University, Alicante, Spain; 3 CIBER of Epidemiology and Public Health (CIBERESP), Barcelona, Spain; 4 Department of Public Health, Institute of Tropical Medicine, Antwerp, Belgium

**Keywords:** Realist evaluation, Intimate partner violence, Primary healthcare teams, Team learning, Health systems, Spain

## Abstract

**Background:**

Despite the existence of ample literature dealing, on the one hand, with the integration of innovations within health systems and team learning, and, on the other hand, with different aspects of the detection and management of intimate partner violence (IPV) within healthcare facilities, research that explores how health innovations that go beyond biomedical issues—such as IPV management—get integrated into health systems, and that focuses on healthcare teams’ learning processes is, to the best of our knowledge, very scarce if not absent. This realist evaluation protocol aims to ascertain: why, how, and under what circumstances primary healthcare teams engage (if at all) in a learning process to integrate IPV management in their practices; and why, how, and under what circumstances team learning processes lead to the development of organizational culture and values regarding IPV management, and the delivery of IPV management services.

**Methods:**

This study will be conducted in Spain using a multiple-case study design. Data will be collected from selected cases (primary healthcare teams) through different methods: individual and group interviews, routinely collected statistical data, documentary review, and observation. Cases will be purposively selected in order to enable testing the initial middle-range theory (MRT). After in-depth exploration of a limited number of cases, additional cases will be chosen for their ability to contribute to refining the emerging MRT to explain how primary healthcare learn to integrate intimate partner violence management.

**Discussion:**

Evaluations of health sector responses to IPV are scarce, and even fewer focus on why, how, and when the healthcare services integrate IPV management. There is a consensus that healthcare professionals and healthcare teams play a key role in this integration, and that training is important in order to realize changes. However, little is known about team learning of IPV management, both in terms of how to trigger such learning and how team learning is connected with changes in organizational culture and values, and in service delivery. This realist evaluation protocol aims to contribute to this knowledge by conducting this project in a country, Spain, where great endeavours have been made towards the integration of IPV management within the health system.

## Background

The life-time prevalence of intimate partner violence against women (IPV)—defined by WHO (World Health Organization) as ‘behaviour within an intimate relationship that causes physical, sexual or psychological harm, including acts of physical aggression, sexual coercion, psychological abuse and controlling behaviours’—ranges from 15% to 71%, with devastating effects on the health and wellbeing of women and children [1-3]. Interventions to prevent IPV, to adequately manage victims of IPV, and to sanction perpetrators need to involve multiple sectors, including legal, social, health institutions, and civil society. Health services can play an important role in the prevention and management of IPV, and there is general consensus on the main actions that the health sector should carry out [4,5]. An expert meeting aimed to provide recommendations for the development of WHO guidelines on health sector response on IPV stated that healthcare services should: ask all women attending services regarding experiences of IPV; stay alert to possible signs and symptoms of IPV; provide healthcare assistance and register all cases; assure women that discomfort and health problems could be related to IPV; inform and orient women on resources available in their communities; ensure privacy and confidentiality; encourage and support women, and respect their own decisions; avoid unsympathetic and blaming attitudes; coordinate with other professionals and institutions; and provide evidence on the magnitude and seriousness of IPV through proper registration and reporting of cases [6].

In recent years, guidelines, norms, and protocols have been produced in many countries in order to incorporate these measures as part of daily health service routines. Social, legal, and other auxiliary services are now more available than twenty years ago, allowing the development of referral networks with health facilities [1,2]. Despite these advances in policy and knowledge generation regarding health systems’ role on IPV prevention and management, there are few examples of countries where the management of IPV has been successfully implemented at the level of service delivery [5,7].

### Innovations, team learning, and health sector response to IPV

Much attention has been given to the diffusion of innovations in healthcare since the publication of Greenhalgh *et al.* in 2004 [8], but little attention has been given to team learning. Numerous studies showed that the extent of adoption of innovations by healthcare teams depends, among other aspects, on the ability of the team to engage in a learning process [9-13]. Team learning is considered as an important factor in adopting the innovation and adapting it to the local context. In this protocol, we conceptualize team learning as an iterative process in which the healthcare team actively adapts and recreates the innovation; ‘a process that includes positive change produced by investment in developing shared insights, knowledge, and skills’ [10].

Team learning involves individual, team, and contextual factors, as well as factors related with the characteristics of the innovation itself [9,10]. Individuals’ characteristics include, *e.g.*, self-efficacy, competence, skills, motivation, or prior experiences. Organizational characteristics refer to the characteristics of the team, *e.g.*, uni- or multi-professionalism, team climate, team identification, team management practices, and leadership characteristics. Contextual factors include societal predisposing influences (*e.g.*, the socio-cultural aspects), and institutional enabling influences (*e.g.*, resource availability, decision spaces) [14,15]. The innovation itself—in terms of perceived benefit, compatibility, complexity, trialability, and observability [10,16]—is also a crucial factor. Moreover, team learning may just be one among other responses of teams to innovations. Health providers may decide to adopt and implement the policy, to adapt it to respond better to suit the needs of patients, or to better suit their own interests or to ignore it [17].

Team learning cannot be explored as a generic entity, but a process highly dependent on the context where it takes place. Regarding IPV, even if research may produce evidence of the beneficial impact on women’s health of the integration of IPV within health services, this knowledge may not be evident for health providers. Integrating IPV within healthcare practices might not be easily compatible with previous practices, in the sense that it involves new skills and procedures (such as empathic interviewing and networking with social services) and may require more time than an ‘ordinary’ medical consultation. Due to the multifaceted nature of IPV, interventions tackling this problem cannot be simple and uniform, but need to involve different sectors. Innovations in IPV management can be tried out by health providers, but lack of self-confidence regarding how to properly address the issue might hinder their willingness to continue using them. Finally, results of the intervention may not be observable, or may not be the ones expected to achieve—*e.g.*, the woman may return to the aggressor. All these factors influence whether PHC teams engage or not in a learning process to adequately manage IPV. Outcomes, in terms of the development of an organizational culture and shared values favorable to IPV management and the actual delivery of IPV management services, will also depend on whether learning processes are generated or not, and on the characteristics of these processes.

When integrating innovations regarding IPV, the way gender is framed has a strong impact on the type and way that IPV management is integrated into health teams and systems [18]. Gender defined as ‘the structure of social relations that centers on the reproductive arena, and the set of practices (governed by this structure) that bring reproductive distinctions between bodies into social processes’ [19] is constructed through relationships that take place at the interpersonal level (gender relations), organizational-institutional level (gender regimes), and broader social level (gender orders) [19,20]. Gender is constructed through the interaction of these levels and is translated into concrete practices within healthcare teams, such as the way men and women work together, or the way health providers approach women who have suffered from IPV.

Despite the existence of ample literature dealing, on the one hand, with the integration of innovations within health systems and team learning, and, on the other hand, with different aspects of the detection and management of IPV within healthcare facilities, research that explores how health innovations that go beyond biomedical issues—such as IPV management—become integrated into health systems and that focus on healthcare teams’ learning processes is, to our best knowledge, very scarce if not absent.

### The policy and interventions to integrate IPV management within the Spanish health system

This study will be conducted in Spain. This country has made remarkable advances in the development of policies against IPV since 1998. ‘The Gender Based Violence Law,’ passed in 2004, represents a progressive and comprehensive development from the two previous action plans (for the 1998 to 2000 and the 2001 to 2004 periods, respectively) [21,22]. On the basis of the law, an array of measures for integral protection against IPV have been implemented, including reforms of the judicial system, extensive training, and the implementation of a comprehensive network of services aimed to protect the rights and safety of women suffering from IPV. Preventive measures directed towards challenging gender inequality at the broader social level have also been established. Despite these noteworthy achievements, IPV remains common, and large differences exist between autonomous regions; *e.g.*, the self-reported one-year-prevalence of IPV ranged from 28.6% in Ceuta and Melilla to 10.7% in Cantabria, Aragon and La Rioja [23].

There are few published studies exploring IPV and the health service response in Spain. The existing research pointed out that: the prevalence of IPV among women who use primary level healthcare facilities is high; women perceive favorably being questioned regarding IPV by the general practitioner; and health services are usually the first institution that women affected by IPV reach [24,25].

The Spanish health system is highly decentralized. Currently, the 17 autonomous regions are in charge of health planning, public health, and management of health services. ‘The Inter-territorial Council of the National Health System’ is the highest authority in decision making regarding health issues in Spain, and representatives from both the national government and the autonomous regions participate in it.

The 1980s health reform and the National Health Law aimed to strengthen primary healthcare. Health delivery was sectorized and in primary healthcare, multidisciplinary work was promoted. However, success of the primary healthcare approach in Spain has been limited, with large variations between autonomous regions. In general, consultation times have decreased, resources remain scarce, bureaucratic ‘red tape’ has not been reduced, and services continue to favour clinical curative activities over promotion and community-based actions [26-28]. This situation constitutes a challenge for the integration of non-biomedical innovations, such as the management of IPV. The current economic crisis and the increasing cost-reduction measures in public services may further decrease the resources devoted to realizing the primary healthcare approach.

The intervention that we aim to evaluate started from the passing of the ‘Gender Based Violence Law in 2004.’ This law, and the most recent ‘Law For Effective Equality Between Women And Men,’ widen and strengthen the role of health services regarding IPV: they are designed to monitor for possible cases of violence, manage them, and engage in a multidisciplinary response coordinating with other institutions and sectors [21,29].

In order to implement the Gender-Based Violence Law in the health sector, a ‘National Commission Against Gender-Based Violence’ (NCAGBV) was created within ‘The Inter-territorial Council of the National Health System.’ The Commission is responsible for monitoring gender-based violence as a public health problem and to improve the prevention, detection, and management of IPV among women attending health facilities. While this description may portray a top-down approach, in reality the implementation of the policy has been less vertical. Indeed, the experience of some regions that started addressing IPV within the health system before the national law was passed was incorporated in the new national policies.

In practice, four main actions have been implemented: development of protocols for a health-care reponse to gender-based violence; training of health professionals; development of information systems; and adapting service delivery [30-36].

Regarding the development of protocols, the NCAGBV developed a common protocol for a healthcare response to gender-based violence published in 2007 and currently under revision [35]. The common protocol helps providers to ensure a proper management of cases of IPV. For proper detection of cases, the protocol encourages general practitioners (GPs) to ask exploratory questions related to IPV during the first consultation. The protocol elaborates on indicators of suspicion of IPV, and provides tips for conducting empathetic interviews; it also explains how to develop a proper assessment of the bio-psycho-social situation of the woman, the type of violence, and the level of risk. The protocol guides the provider through the intervention process, reminding him/her of the relevant matters that should be addressed [33,35]. The conflict between the providers’ obligation to report (in Spain reporting IPV is mandatory for health providers) and the need to respect women’s autonomy is mentioned in the protocol. Due to the participation of representatives of the autonomous regions in the NCAGBV, earlier experiences and protocols from the regions have also informed the elaboration of the national protocol.

Regarding training of health professionals, a working group for training and supervising health professionals on IPV has been created within NCAGBV, and each autonomous region has developed ways to support training. A guide with the basic contents that all training processes should include has been published [36]. Training has targeted providers at the first level and has taken a variety of forms, *i.e.*, training on empathetic interviewing and screening, sensitizing on gender equality, and trainer improvement programs. Some ongoing impact evaluations show promising results: training improves health staff awareness and self-confidence, and increases the number of cases detected. Training also enhances providers’ knowledge and appreciation of other available resources (social, emergency, legal), and may strengthen coordination between different health services, as well as interdisciplinary coordination [32-34].

Regarding information systems, the NCAGBV has agreed upon 18 common indicators (although recently they were reduced to 11) in order to improve the quality of information gathered regarding IPV. These indicators are used to measure the prevalence of IPV detected within health facilities and allows disaggregation by the type of violence, women’s characteristics, and type of service. They also provide information about the characteristics of the care provided at the health facility, and the prevalence and type of referrals. How these or other indicators are used to monitor and support the work of healthcare teams on IPV management remains less clear [33,34].

Regarding service delivery, it is assumed that all the three interventions described above support the improvement of service delivery for IPV. Additionally, many autonomous regions have developed separate multisectorial plans and pathways to support an interdisciplinary response to women suffering from IPV, connecting the health services with other sectors. Some autonomous regions have, for example, included special measures such as the inclusion of IPV management within the essential components of the PHC portfolio, or the implementation of screening for IPV within PHC services or antenatal care services [30-34].

We argue that all these actions had the potential to generate learning processes within primary healthcare teams. However, it can be assumed that not all primary healthcare teams generated the same learning processes, and consequently, they introduced the management of IPV to a different extent or in diverse ways. These differences might be due to regional factors—due to decentralization of healthcare services, differences exist between autonomous regions in terms, *e.g.*, of the development of regional protocols, the way service is delivered, training schemas, and information systems—and factors that pertain to the team itself.

In this paper, we present the protocol of an evaluation of the integration of IPV management within the Spanish health system, focusing on service delivery within primary healthcare facilities. The study aims to ascertain: why, how, and under what circumstances primary healthcare teams engage (if at all) in a learning process to integrate IPV management in their practices; and why, how, and under what circumstances team learning processes lead to the development of organizational culture and values regarding IPV management, and the delivery of IPV management services. The study will take a realist evaluation approach, exploring the mechanisms through which primary healthcare (PHC) teams learn to integrate IPV management.

## Methods

### Realist evaluation

In this study, we will use a realist evaluation approach. Realist evaluation is a type of theory-driven evaluation well-suited for evaluating complex intervention [37]. Based on Pawson and Tilley’s work, it looks beyond the effect of individual factors to include assessing the mechanisms of change that, triggered by the intervention in particular contexts, led to the outcomes [37-39].

Realist evaluation starts with the formulation of a preliminary middle-range theory (MRT) that connects context, mechanisms, and outcomes—potential ‘CMO configurations.’ These initial MRTs are formulated based on previous research, and/or on the knowledge and experiences of stakeholders involved in the design of the intervention evaluated [37,38,40]. They serve to guide the data collection process. Data analysis leads to refining the initial MRT. The refined MRT provides plausible explanations of why, how, and under what circumstances the intervention triggered certain mechanisms that led to certain outcomes. The refined MRT does not constitute the end of the process, but is the starting point for a new cycle of realist evaluation. In analogy with theory-driven evaluation [41,42], it is useful to consider that the MRT has an action model and a causal model component. The first deals with assessing the implementation and outcomes, the latter with the causal explanations.

A critical element in realist evaluation is that of mechanisms. Mechanisms intermediate between the concrete components of the interventions and the outcomes. According to Pawson and Tilley a mechanism is ‘not a variable but an account of the behaviour and interrelationships of the processes that are responsible for the change’ (Cited by [43]). Other authors have argued that elucidating mechanisms could be a useful way to bridge the gap between theory building and practical recommendations [44,45]; if we are able to identify the mechanisms that lead to positive change, they can guide scaling-up processes.

### Conceptual framework

**Figure 1:**
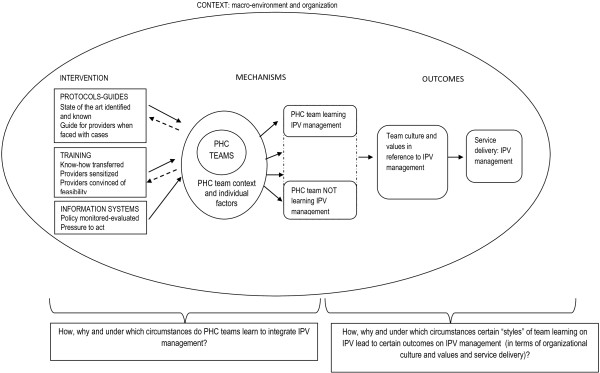
A conceptual framework for analyzing the process of team learning of IPV management within primary healthcare teams.

Combining the concepts of team learning and the multipolar performance framework for assessing the dynamics of performance of healthcare organizations [46], we came up with a conceptual framework to analyze how learning of IPV management is generated within primary healthcare teams (see Figure 1).

The framework represents how the intervention—through the development and dissemination of protocols, training of health professionals, and the implementation of an information system to report all cases of IPV—triggers (or not) processes of team learning on IPV management within PHC teams, and leads to improved service delivery, whereby it is assumed that the organizational culture and values will change in favor of providing high-quality IPV management.

Teams’ responses to the intervention may differ. Some teams will totally ignore the policy, while others will engage eagerly in team learning. In between these two extremes, a variety of responses are possible (represented by the dotted lines in Figure 1). The learning styles are also likely to vary. Furthermore, this process does not take place in a vacuum, but is strongly influenced by the interaction of the team with the context where these teams work. This includes the broader social context (*e.g.*, society perception of IPV and degree of tolerance), the organizational context (*e.g.*, health system organization, the extent that it has incorporated IPV as a public health problem) and the team context (*e.g.*, team climate, team previous learning experiences, the individual characteristics of the team members). Moreover, even if this seems to be a top-down process, where interventions are delivered from the regional level to the health teams, there is likely to be room for PHC teams to influence the intervention above their specific health service—*e.g.*, by being invited to train other health professionals or to participate in the development of guidelines.

### Method and steps

This study follows the steps described by Pawson, and applied *e.g.*, by Prashanth *et al.*, Rycroft-Malone *et al.*, and Ranmuthugala *et al.* in their recently published realist evaluation protocols [47-49]: eliciting the implicit theories; formulate initial MRT; testing initial MRT; and specification. A summary of the steps, the objectives of each step, and the methods for data collection that will be used can be found in Table 1.

**Table 1:** Four-step approach to realist evaluation of primary healthcare team learning on IPV management

**Step**	**Activities**	**Analysis**
Step 1. Eliciting the implicit theories	• Review of policies, programs, unpublished reports and statistical reports regarding the intervention delivered.	Qualitative-thematic analysis driven by the conceptual framework presented in Figure 1.
	• Semi-structured interviews with stakeholders and providers involved in the intervention.	
Step 2. Formulation of the initial middle-range theory	Contrasting and complementing implicit theories with findings from the literature review	Revise and propose initial middle-range theory configurations in the light of the information collected during Step 1.
Step 3. Testing the initial middle-range theory	• Select cases for testing of the initial MRT.	Qualitative- thematic analysis of collected data using the CMO approach
	• Case studies with quantitative and qualitative data collection	
Step 4. Specification	Refine the initial middle range theory in the light of the retained CMO configurations	Draft refined middle-range theory that explains how and under which contextual factors primary health care teams learn to provide adequate IPV case management

Realist evaluation is method neutral, and authors using this approach have employed different designs and methods for data collection and analysis [39,50]. In this study, we will use the multiple case study design [51]. Data will be collected from selected cases (primary healthcare teams) through different methods: individual and group interviews, routinely collected statistical data, documentary review, and observation. Cases will be purposively selected in order to enable testing the initial MRT. After in-depth exploration of a limited number of cases, additional cases will be chosen for their ability to contribute to refining the emerging MRT.

### Step one: eliciting the implicit theories

In this first step, we will elicit the implicit program theories that are held by the policy designers at national level and implementers at the regional and district level. Besides national level stakeholders, semi-structured interviews with be held with stakeholders at the political and managerial level of the regional health system, professionals involved in training of health providers on IPV, the development of guidelines regarding IPV, the development and implementation of IPV information systems, primary healthcare managers at district level, and representatives of other sectors working against IPV within the district.

Interviews will be fully transcribed and imported into Open Code 3.1 for managing the coding process [52]. Secondary data from policies and documents will also be coded, and emerging codes will be grouped into themes following thematic analysis [53], driven by our conceptual framework.

### Step two: formulation of the initial MRT

In this step, the literature on IPV and team learning will be further reviewed and findings will serve to contrast or complement the implicit program theory emerging from step one. Edmondson *et al.*, in their review about team learning research, stresses that defining team learning is strongly dependent on the context and the intervention to be learned, and highlights the need to conduct research that considers these specificities [10]. We surveyed the literature dedicated to locating published studies on primary healthcare team learning on IPV, but none were found. Consequently, we reviewed both the literature on team learning and the literature on health sector responses on IPV, focusing on primary healthcare settings, in order to develop a preliminary list of elements that might be relevant for team learning on IPV (see Table 2).

**Table 2:** Preliminary list of potential elements that may play a role in initiating and maintaining team learning on IPV within primary health care teams: context, mechanisms, and outcomes

**Realist evaluation concepts**	**Potential elements**
**Context factors**	Policies and procedures in place regarding IPV
Outer context	Social environment regarding IPV
	Existence of coordinated multi-sectorial responses against IPV

Organizational context	IPV perceived as important within the health system
	Policies and procedures in place regarding health sector response to
	IPV: protocols, IPV in
	plans, IPV in service portfolio
	Knowledge transfer efforts on IPV
	Accountability and monitoring systems regarding IPV established
Team context	Team climate and identity
	Previous team learning processes
	Group processes within team meetings and outside team boundaries
	Power differences within teams
	Leader behavior
	Quality of team interpersonal processes
	Team composition – interdisciplinarity, professional guilds –, size and stability
**Individual factors**	Individuals’ perceptions regarding IPV
	Individuals’ characteristics: feasibility, can-do attitude
	Individuals’ perceptions of the interventions regarding IPV to be implemented: perceived benefit, compatibility, complexity, trialability and observability
**Mechanisms**	Team psychological safety to learn IPV management
At team level	
	Team learning behavior on IPV management
	Team reflexivity on IPV management
	Team’s approach to gender and IPV
At individual level	Integration-and-learning perspective: creating shared learning goals on IPV management
	Self-confidence generated within the team to try new things; self-Efficacy Commitment (will-do)
**Outcomes**	Health professionals sensitized regarding IPV and working together in this issue
Team culture and values in reference to IPV management	Team has ways for continuous knowledge transfer on IPV management and engaging newcomers
Service delivery-operational management	Health teams adequately managing cases of IPV (according to WHO and National guidelines)
	Cases of IPV at PHC facilities are appropriately reported and referred to relevant services
	Prevention and promotion activities regarding IPV are carried out
Service delivery-service provision	Health teams provide more appropriate services to more women victims of IPV

### Step three: testing initial MRT

For empirical testing of the MRT developed during step two, selected cases will be explored in depth. The cases will be purposively selected based on a theoretical replication argument [48], meaning that cases will be selected on their potential to provide contrasting contexts and/or outcomes, the analysis of which contributes to the refinement of the MRT. We will start with an in-depth exploration of a small number of cases in one specific region/district. We envisage that some cases would constitute an example of outstanding achievements on IPV management, while others would be average primary healthcare centres in terms of IPV management. This contrastive approach will enable us to explore the role of individual and team factors in translating regional-district interventions into outcomes. This design has been used previously in realist evaluation [40]. All cases of this study will be primary healthcare facilities serving towns and cities of between 20,000 and 100,000 people in order to ensure that selected facilities are located in settings with access to other services supporting women suffering from IPV.

From each case, information will first be collected concerning the actual implementation of the intervention to assess the relevance of the program design and the degree of implementation. This constitutes an assessment of the action model—whether the intervention was delivered to the cases as it was planned and envisaged or not—and evaluates whether failure of the intervention could be due to implementation failure. Next, we will assess the initial causal model and assess the contextual factors, mechanisms, and outcomes and the interaction between them.

In each case, data will be collected through: review of documents and statistical reports; audit of clinical records; observation of consultations and team meetings; in-depth interviews with members of the team; focus group discussions with the teams; and in-depth interviews with persons responsible for social services related to IPV in the area of influence of the case. The document review will include evaluation reports, existing local guidelines or protocols, and case-specific routine data related to IPV (the 11 common indicators and other data) during the past years. Clinical records of consultations with women during the previous year will be revised in order to ascertain whether the possibility of suffering from IPV is assessed or not. The clinical records of cases of violence will be further analyzed to assess how the providers dealt with the case and whether they covered all aspects.

The observations will assess: whether national and/or regional laws, protocols and guidelines on IPV are present, available, and used; the presence of promotional material on IPV in the waiting areas and consultation rooms; the interaction during medical consultations with women users; whether active inquiry for IPV is conducted during the consultation; the existence and use of instruments for IPV screening; the adequacy of the intervention when IPV is detected; and the interactions between healthcare team members during daily activities and meetings, and others that may emerge.

During the interviews, an open guide will be used and issues explored will include: the perceptions on IPV and gender; the participant’s experience in detecting and managing cases of IPV; the process of integrating IPV within the healthcare service; training activities, participation, and the participant’s perceptions regarding the training; the use of protocols and guidelines and perceptions; the reporting systems; the active inquiry for IPV during consultations (in case the participant is a provider); the intervention in cases of IPV; the relationships between team members; and the support that is received and how such support is perceived. Focus group discussions will explore similar issues, but will pay specific attention to the interaction between team members, the relationships and dynamics, and gender regimes. Finally, interviews with providers of social services related to IPV will enable insights in the inter-sectoral collaboration at the case study site and in how the integration of IPV management by the healthcare is perceived by the involved external actors. The instrument to guide the data collection from the selected healthcare teams can be found in Additional file 1.

Interviews and focus group discussions will be conducted in Spanish, digitally recorded, and fully transcribed verbatim. The transcripts and the reports from the observations will be imported into Open Code 3.1, a software program for managing qualitative data [52]. Initial codes will be developed from the transcripts. These codes, together with the coding of secondary data (*e.g.*, prior evaluations) will be grouped into themes following thematic analysis [53]. The MRT and its inherent CMO configurations developed during step two will guide the thematic analysis, but we will remain open to new emerging themes.

### Step four: specification

In the light of the findings from step three, the initial MRT will be refined so as to reflect the results of the case: emerging conjectural CMO configurations will be assessed against the data and the retained CMO configurations compared with the initial MRT. The resulting refined MRT will reflect the insights gained from the case.

### Ethical considerations

The study has been approved by the Ethical Committee of the University of Alicante. Each participant in the study will be asked for a written informed consent previous to conducting the interviews and during the observations. In any subsequent report or publication, information that could identify the respondents will not be used. Also for the review of clinical records, any identifier will be removed. Results will be fed back to the health authorities in the autonomous regions and to the staff of the case study sites via a report in Spanish.

## Discussion

This paper describes a protocol that uses a multiple case-study design to understand how, why, and when primary healthcare teams learn to integrate IPV management.

Over recent years, realist evaluation has been gaining increased recognition within health systems research [45,47,48,50,54-59]. For health systems research, it is important to assess interventions not only under controlled conditions (efficacy), but especially under real circumstances where contextual factors play an important role (effectiveness). Realist evaluation, instead of controlling for contextual factors, explores them in interaction with the intervention, the outcomes, and the mechanisms. It tries to find demi-regularities that help explain why, how, and when processes take place. This approach is suitable for exploring complex interventions, such as the integration of socio-sanitary programs within healthcare services [45].

Other authors have warned regarding the challenges associated with realist evaluations [39,47,59]. In the development of this protocol, many of these challenges became clear. The first challenge emerged while developing the potential CMO configurations for further exploration (as on Table 2): on which basis should the MRT be developed when evidence is scarce? Indeed, on one hand, published research on IPV management within a health system’s approach is scarce, and research focusing on team learning in IPV management even more so. On the other hand, programs and interventions carried out in the Spanish Health System with this aim have been numerous and diverse, but the program theories that drove these interventions were not made explicit nor published. That meant that our preliminary MRT will emerge from the available literature and interviews with stakeholders and should really be considered as preliminary.

The second challenge refers to measuring the effect of the intervention on team culture and values and service delivery in a feasible way. Regarding team culture, we decided to focus on collecting qualitative data through observation and in-depth interviews, because we consider it more meaningful than a quantitative survey on attitudes and practices. Regarding service delivery, we decided to use routinely collected data, observations, interviews, and the audit of clinical records. Some challenges emerge with the latter; *i.e.*, the quality of routinely collected data may differ from setting to setting, and safety and privacy issues may make observation of interactions between providers and women reporting IPV impossible in certain cases. The resulting information gaps will be filled because we have planned to collect information on the outcomes from a number of different sources.

A third challenge refers to the fact that the interventions we intend to evaluate were initiated years ago and that they have evolved over time. To document these changes in the intervention is challenging, but this has also been done before in other studies using realist evaluation [57]. Moreover, it has been argued that realist evaluation is well suited for examining the diverse components of a program in a contextualized way [47]. This is especially relevant for a multi-faceted intervention such as the one evaluated in this study.

A fourth challenge relates directly to the current situation of economic crisis and cost-reduction measures in the Spanish healthcare and social system. When this research study was initially planned, Spain was allocating a large number of resources to fight IPV, and the Spanish healthcare system was one of the most comprehensive in Europe. Currently, investment in social programs and in the health system has been drastically reduced [60], with significant variation from region to region. These changes in the context and the intervention will need to be taken into account during the implementation of the protocol.

Despite the existence of ample literature on IPV and health, and a general consensus on the importance of a health sector response in the fight against IPV, few health systems have institutionalized IPV detection and management in a successful way. Evaluations on health sector responses to IPV are scarce, and even fewer focus on why, how, and when the healthcare services integrate IPV management. There is a consensus that healthcare professionals and healthcare teams play a key role in this integration, and that training is important in order to realize changes. However, little is known about team learning on IPV management, both in terms of how to trigger such learning and how team learning is connected with changes in organizational culture and values, and in service delivery. This realist evaluation protocol aims to contribute to this knowledge by conducting this project in a country, Spain, where great endeavours towards the integration of IPV management within the health system have been carried out.

## Competing interests

The authors declare that they have no competing interests.

## Authors’ contributions

IG participated in the design of the protocol, wrote the first draft of the manuscript, and was mainly responsible for incorporating co-authors suggestions and changes. CVC, AKH, and MSS participated in the design of the protocol, and contributed to the drafts of this manuscript. BM and GK revised the first drafts of the protocol and made significant contributions for its improvement. All authors read and approved the final manuscript.

## Supplementary Material

Additional file 1 **Appendix 1. **Frame to guide data collection from the cases, following a realist evaluation approach. Click here for file
