# Supplementary material for: How do primary health care teams learn to integrate intimate partner violence (IPV) management? A realist evaluation protocol
Source: Implement Sci. 2013 Mar 23;8:36. doi: 10.1186/1748-5908-8-36 (PMC3617002; doi:10.1186/1748-5908-8-36)
Supplement: Additional file 1 — Appendix 1. Frame to guide data collection from the cases, following a realist evaluation approach. [file 1748-5908-8-36-S1.pdf]

Additional File 1. Frame to guide data collection from the cases, following a realist evaluation approach.

**ENABLING POLICY ENVIRONMENT AND NETWORKS**			
**Criteria**	**Indicator**	**Source and method for collecting data**	**CMO in focus**
Coordination	Exists figure of coordinator/responsible for IPV within health service	Interviews	Outcome
Relationship with higher levels	Has somebody in the health center participated in the working groups of the NCAGBV?	Documentary review, interviews	Context
	Relationship with regional representatives of the groups or other persons responsible for IPV?	Documentary review, interviews	Context
Care pathways for abused women available	Relationship-networks with other resources for abused women in the neighborhood and their response	Interviews with HF and other resources, documentary review	Context, Mechanisms
	Local pathways for abused women available and used in HC	Documentary review, interviews, observation	Outcome

**TEAM CULTURE AND VALUES**			
**Criteria**	**Indicator**	**Source and method for collecting data**	**CMO in focus**
Attitudes and values regarding IPV	PREMIS questionnaire- knowledge and attitudes	Interviews	Outcome

TRAINING OF HEALTH PROFESSIONALS			
**Criteria**	**Indicator**	**Source and method for collecting data**	**CMO in focus**
All staff involved in training	Training activities in which staff have participated (type, duration, etc)	Interview	Context (Intervention)
Supervision and reinforcement	Periodic meetings that discuss cases of IPV	Interviews & observation & reports (minutes of meetings)	Outcome
Perceptions on training process:	Logistic aspects: easy to attend? Well rewarded/acknowledged?	Interviews	Context (Intervention), Mechanisms
	Training- contents: which aspects were most interesting? Which aspects most conflictive?	Interviews	Context (Intervention), Mechanisms
	Training-applicability: Which aspects more relevant for their work?	Interviews	Context (Intervention), Mechanisms
	Training-quality: Quality of trainers and training?	Interviews	Context (Intervention), Mechanisms
	Training- support: evaluate monitoring and support provided, continuous training, ability to try things	Interviews	Context (Intervention), Mechanisms
	Training-implementation: the process from attending training to implementing changes	Interviews	Mechanism

ACCOUNTABILITY AND MONITORING			
**Criteria**	**Indicator**	**Source and method for collecting data**	**CMO in focus**
Monitoring system that provides data on number of cases	Common indicators 1-11 from 2008 until 2012	Collected data in HC; measure whether data are collected and changes through time	Outcome
	PREMIS questionnaire-cases attended	Questionnaire applied to all providers	Outcome
	Other indicators related to IPV collected in the HC	Collected data in HC; measure whether data are collected and changes over time	Outcome
Debriefing support	Does it exist ways for de-breefing support?	Interviews	Outcome
System to learn from women's experiences of the service	Does it exist ways to collect information on women's experiences of the service?	Interviews & Observation (existence of mechanisms or not and women's view of service)	Outcome

PROTOCOLS AND GUIDELINES			
**Criteria**	**Indicator**	**Source and method for collecting data**	**CMO in focus**
Clinical guidelines for responding to IPV in place and implementation monitored	Protocol and/or guidelines available and used or own protocol elaborated	Interviews & Observation	Outcome
	Procedures to monitor adequate implementation of IPV management	Interview & Observation & Documentary review (minutes)	Outcome, Mechanisms
Health systems and services	Providers during the consultation document what the woman says	Interview & Observation & Audit of clinical records	Outcome
	Providers during the consultation validate the women's experiences	Interview & Observation	Outcome
	Providers have non-judgmental attitudes	Interview & Observation	Outcome
	Providers give medical treatment and inform about crisis center and long-term services	Interview & Observation & audit of clinical records	Outcome
	Providers support safety planning	Interview & Observation & audit of clinical records & Common Indicator	Outcome
	Providers refer to other services	Interview & Observation & audit of clinical records & Common Indicator	Outcome

Psychosocial and emotional support	Providers listen and assess the risks	Interviews & Observation & audit of clinical records	Outcome
	Providers do not contact the woman's partner	Interviews & Observation & audit of clinical records	Outcome
Non-negotiable issues	Provider reassures the woman that confidentiality will be safeguarded	Interviews & Observation	Outcome
	Medical records are kept confidential and safe	Interviews & Observation	Outcome
"Screening"	Screening in ANC	Interview & Observation & Documentary review (records, screening tools)	Outcomes
	Screening in abortion care	Interview & Observation & Documentary review (records, screening tools)	Outcomes
	During first consultation with women, exploratory questions to assess the existence of IPV are introduced	Interview & Observation & records (audit clinical records)	Outcomes

PREVENTION, PROMOTION AND WORK WITH WOMEN IN VULNERABLE SITUATIONS			
**Criteria**	**Indicator**	**Source and method for collecting data**	**CMO in focus**
Prevention and promotion activities carried out by the HC team	Any activities carried out aimed for prevention of IPV	Interviews & Observation & documentary review	Outcomes
	Any activities focusing on women in situation of vulnerability	Interviews & Observation & documentary review	Outcomes

OTHERS			
**Criteria**	**Indicator**	**Source and method for collecting data**	**CMO in focus**
	Any prize or acknowledgment received	Interviews & Observation & documentary review	Outcomes
	Reports of previous evaluations	Interviews & Documentary review	All
	Research conducted	Interviews & Documentary review	All
	Perception of other actors dealing with IPV in the neighborhood regarding the HC management of IPV	Interviews	All

CMO in focus: Intervention, context (local) and Outcomes. Mechanisms are developed through the interaction of all of them, and the exploration of all will add to defining mechanisms. Context here refer to the local context where the HF is located and also on how the HF perceives its relationship with the regional and national context (collected in the previous study)
